# Role and molecular mechanism of *Salvia miltiorrhiza* associated with chemical compounds in the treatment of diabetes mellitus and its complications: A review

**DOI:** 10.1097/MD.0000000000037844

**Published:** 2024-04-19

**Authors:** Jiajie Li, Jinxing Liu, Weibing Shi, Jinchen Guo

**Affiliations:** a School of Integrated Chinese and Western Medicine, Anhui University of Chinese Medicine, Hefei, Anhui, PR China; b The First Affiliated Hospital of Anhui University of Chinese Medicine, Hefei, Anhui, PR China; c School of Traditional Chinese Medicine, Anhui University of Chinese Medicine, Hefei, Anhui, PR China.

**Keywords:** chemical compounds, diabetes mellitus, pharmacological effects, *Salvia miltiorrhiza*

## Abstract

Diabetes mellitus (DM) is one of the most prevalent diseases worldwide, greatly impacting patients’ quality of life. This article reviews the progress in *Salvia miltiorrhiza*, an ancient Chinese plant, for the treatment of DM and its associated complications. Extensive studies have been conducted on the chemical composition and pharmacological effects of *S miltiorrhiza*, including its anti-inflammatory and antioxidant activities. It has demonstrated potential in preventing and treating diabetes and its consequences by improving peripheral nerve function and increasing retinal thickness in diabetic individuals. Moreover, *S miltiorrhiza* has shown effectiveness when used in conjunction with angiotensin-converting enzyme inhibitors, angiotensin receptor blockers (ARBs), and statins. The safety and tolerability of *S miltiorrhiza* have also been thoroughly investigated. Despite the established benefits of managing DM and its complications, further research is needed to determine appropriate usage, dosage, long-term health benefits, and safety.

## 1. Introduction

Diabetes mellitus (DM) affects approximately one in twenty people and remains a leading cause of disability.^[1,2]^ There were 537 million adults (aged 20–79 years) with diabetes worldwide in 2021.^[3]^ A bulk of studies have investigated the molecular mechanisms involved in the onset and progression of DM, as well as the potential therapeutic options. There is growing evidence showing a close relationship between inflammation and oxidative stress with the onset and progression of DM. Oxidative stress is considered to be a major contributor to type 2 diabetes mellitus (T2DM) as pancreatic β cells are susceptible to reactive oxygen species due to their low antioxidative capabilities.^[4]^ In addition, inflammatory factors can promote insulin resistance via modulation of β-cell function and interaction with insulin signaling.^[5]^ While treatments are available, effectively managing DM and its associated complications remains challenging.^[6]^ *Salvia miltiorrhiza*, also named blood ginseng or red ginseng, is mainly cultivated in China and other Asian countries including Japan, and Korea.^[7]^ Considering that the primary efficacy of *S miltiorrhiza* is to promote blood circulation, remove blood stasis as well as calm the mind, *S miltiorrhiza* has been widely utilized in the treatment of cardiovascular diseases, and a variety of inflammation-related diseases.^[8]^ Traditional Chinese medicine, including the use of *Salvia miltiorrhiza*, has been widely employed in DM treatment. Recently, the potential therapeutic effects of *S miltiorrhiza* on DM and its complications have drawn increasing interest.^[9]^ This review aims to provide an overview of current developments in utilizing *S miltiorrhiza* for treating DM. It covers the disease pathogenesis, complications, pharmacological actions, clinical applications, benefits, drawbacks, and future prospects for research and application. A comprehensive understanding of *S miltiorrhiza* potential as a DM treatment could facilitate the development of novel therapeutic approaches for this condition.

## 2. The overview of DM

### 2.1. Definition and classification of DM

The illness known as DM is caused by impaired or dysfunctional pancreatic islet β-cells. Numerous studies have shown that type 1 diabetes mellitus (T1DM) is more susceptible to genetic influences, while T2DM is largely influenced by lifestyle factors.^[10]^ T1DM, an ongoing autoimmune condition characterized by hyperglycemia, is one of the most common endocrine and metabolic diseases in children.^[11]^ The detection of autoantibodies is considered a biomarker for presymptomatic T1DM.^[12]^ It is noteworthy that T2DM affects more than 85% of people with diabetes. Unhealthy lifestyle choices such as consuming high-fat meals and leading a sedentary lifestyle have contributed to the increased prevalence of T2DM. Fasting plasma glucose levels, HbA1c levels, and oral glucose tolerance tests can all be used to confirm the diagnosis.^[1]^

### 2.2. Influence of long-term complications

DM complications are commonly divided into microvascular complications and macrovascular disease. Microvascular complications include diabetic retinopathy, diabetic nephropathy, peripheral sensorimotor neuropathy, and autonomic neuropathy. Diabetic retinopathy affects over 80% of patients with DM and is a leading cause of visual impairment.^[13–16]^ The compensatory proliferation of retinal vessels stimulated by DM leads to protein and blood leakage, which may ultimately result in blindness.^[17,18]^ Diabetic nephropathy is diagnosed when urinary albumin excretion increases without other renal conditions. Insulin-independent glucose uptake by kidney cells promotes glucose metabolism through non-glycolytic pathways, causing mitochondrial dysfunction, oxidative stress, and inflammation.^[19,20]^ Peripheral sensorimotor neuropathy and autonomic neuropathy are caused by damage to Schwann cells, leading to demyelination.^[21,22]^

Coronary cardiac disease, cerebrovascular accident, and peripheral artery disease are the 3 main types of macrovascular disorders. Patients with diabetes are more likely to develop coronary heart disease.^[23]^ Coronary heart disease is a heart condition caused by insufficient blood supply to the coronary arteries, which may result in angina, myocardial infarction, and heart failure. Additionally, diabetes patients are at greater risk of cerebrovascular diseases,^[24]^ including ischemic and hemorrhagic strokes, which can lead to stroke, cognitive impairments, and hemiplegia. Peripheral arterial disease is also common in diabetes patients,^[25]^ affecting the arteries in the lower extremities and other limbs. It can cause inadequate blood supply to the lower limbs, leading to intermittent claudication, ulcers, and gangrene. The occurrence of these complications in diabetes patients is closely related to prolonged hyperglycemia. High blood glucose levels can damage endothelial cells, promote atherosclerosis formation, and subsequently result in vascular stenosis and obstruction.^[26,27]^

The current clinical approaches for treating diabetes patients with concomitant macrovascular disease mainly involve the following 3 aspects:

(1) Medication therapy: Controlling the underlying pathological processes often requires medication.^[28]^ Commonly used medications include:
  a. Antiplatelet drugs, such as aspirin, to prevent thrombus formation and cardiovascular events.
  b. Angiotensin-converting enzyme inhibitors and angiotensin receptor blockers (ARBs), used to control hypertension and reduce cardiac and vascular burden.
  c. Cholesterol-modulating drugs, such as statins, used to lower cholesterol levels in the blood and reduce the risk of atherosclerosis.
  d. Blood glucose control medications, which help reduce the damaging effects of hyperglycemia on blood vessels by controlling blood sugar levels.
(2) Lifestyle management^[29]^: Adopting a balanced diet, limiting the intake of high-fat, high-cholesterol, and high-salt foods, and increasing the consumption of vegetables, fruits, and whole grains is crucial for preventing and treating complications in diabetes. Regular exercise, such as brisk walking, swimming, and cycling, helps control weight, lower blood pressure, and improve blood circulation. Smoking cessation is also essential as tobacco use further impairs vascular function and increases the risk of cardiovascular events.
(3) Surgical intervention^[30]^: For patients with severe diabetes and concomitant large vessel diseases, surgical interventions may be necessary to improve vascular lesions or restore blood flow. Common surgical interventions include:
  1. Coronary revascularization techniques, such as percutaneous coronary intervention and bypass grafting of the coronary arteries, are used to restore normal blood flow to the coronary arteries.
  2. Vascular interventions, such as angioplasty or vascular bypass surgery, are employed to improve blood circulation in peripheral arterial disease.

### 2.3. Mechanisms/pathophysiology of DM

#### 2.3.1. T1DM

The human leukocyte antigen (HLA) class II haplotypes, such as HLA-DR3-DQ2 and HLA-DR4-DQ8, are the main genetic risk factors for T1DM on chromosome 6.^[31–33]^ The presence of both HLA-associated risks and autoantibodies targeting β-cells increases the likelihood of developing T1DM, as both haplotypes are also significant risk factors for the production of these antibodies. Furthermore, studies have demonstrated that HLA-associated genetic risk factors are associated with the type of autoantibody that appears first.^[34–36]^ In contrast to insulin autoantibodies, individuals with the HLA-DR3-DQ2 haplotype are more likely to initially acquire GAD65 autoantibodies.^[37]^ Additionally, more than 50 non-HLA genetic variants that contribute to the risk of T1DM have been identified through genome-wide association studies.^[38]^

#### 2.3.2. T2DM

The development of T2DM is attributed to various factors, including changes in the microbiome, immune dysregulation, and inflammation, which have the potential to be targeted therapeutically.^[39]^ Furthermore, multiple studies have highlighted several mechanisms that contribute to the rapid progression of the disease. These processes include sodium-glucose co-transporter-2 hyperexpression, sophisticated end product of glycation production, coagulation disorders, enhanced platelet reactivity, and impaired endothelial function.^[40–42]^

## 3. Chemical composition and pharmacological effects of *S miltiorrhiza*

Traditional Chinese medicine has employed the plant *S miltiorrhiza* for generations to treat a variety of illnesses. Understanding *S miltiorrhiza* chemical makeup and pharmacological impact has advanced significantly in recent years^[43–45]^ (Table 1).

**Table 1:** Chemical composition and pharmacological effects of *S miltiorrhiza*.

Compound name	Bioactive activities	References
Tanshinones	Antioxidant activity	^[46,47]^
	Anti-inflammatory activity	^[48,49]^
	Vasodilation activity:	^[50]^
	Anti-thrombotic activity	^[51,52]^
	Antitumor activity	^[31,53]^
	Effects on cardiovascular and cerebrovascular diseases	^[54]^
	Antidiabetic effects	^[55]^
	Hepatoprotective effects	^[56,57]^
	Effects on neurodegenerative diseases	^[58]^
*S miltiorrhiza* polysaccharides	Antioxidant activity	^[59,60]^
	Anti-inflammatory activity	^[61]^
	Cardiovascular protection	^[62,63]^
	Antitumor activity	^[64,65]^
	Antidiabetic effects	^[66,67]^
	Immunomodulatory effects	^[68]^
	Hepatoprotective effects	^[44,69,70]^
	Neuroprotective effects	^[71,72]^
Salvianolic acid compounds	Antioxidant effect	^[73]^
	Anti-inflammatory effect	^[74]^
	Cardiovascular protection	^[75–78]^
	Antiplatelet aggregation effect	^[77]^
	Antitumor effect	^[79]^
	Antibacterial effect	^[80]^
	Immunomodulatory effect	^[81,82]^
	Anti-aging effect	^[83]^

### 3.1. Tanshinones (Tans, Tan IIA, Tan I, cryptotanshinone and dihydrotanshinone)

#### 3.1.1. Multiple mechanisms of pharmacological effects of Tan compounds

**Figure 1.**
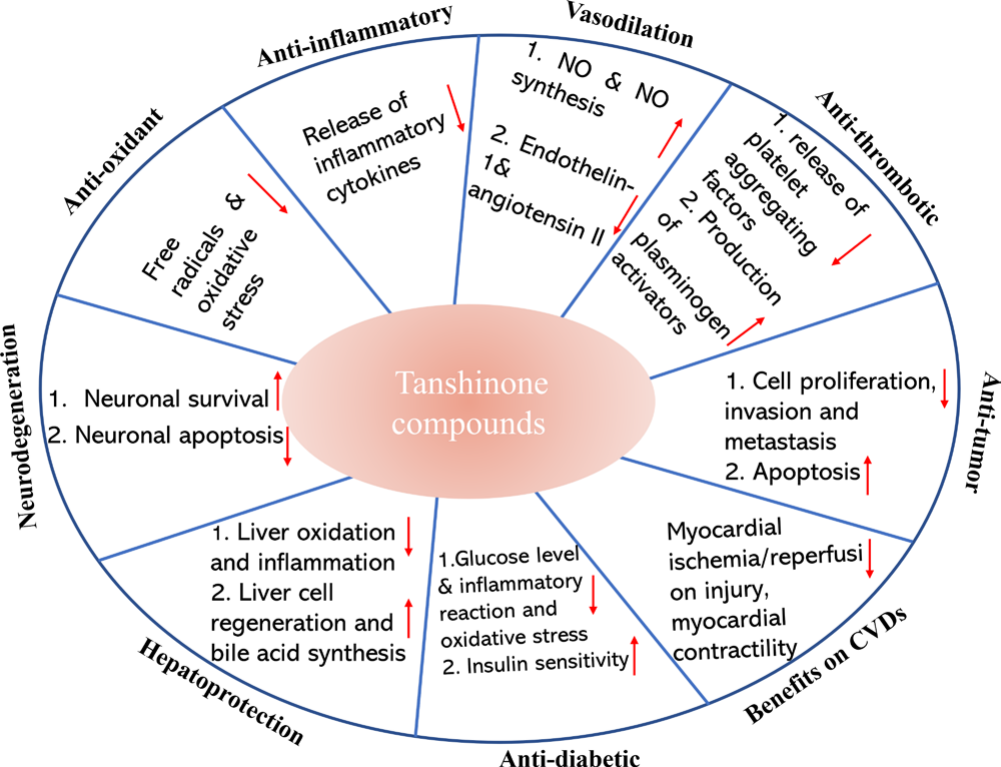
Overview of the pharmacological effects of Tan compounds.

One of the main lipophilic substances obtained from *S miltiorrhiza* root juice is tans. The pharmacological effects of Tan compounds involve multiple mechanisms (Fig. 1). Here are the main mechanisms of action:

1. Antioxidant activity: Tan compounds possess significant antioxidant activity, enabling them to scavenge free radicals and inhibit oxidative stress reactions. They regulate the balance of oxidation-reduction, reduce oxidative damage, and minimize the generation of intracellular peroxides, thereby protecting cells against oxidative stress. Numerous studies have shown that Tan IIA increases the amounts of antioxidant enzymes such as glutathione peroxidase (GPx), superoxide dismutase (SOD), and catalase to reduce oxidative stress.^[46,47]^
2. Anti-inflammatory activity: Tan compounds exhibit regulatory effects on inflammatory responses. They can suppress the release of inflammatory cytokines such as tumor necrosis factor-alpha (TNF-α), interleukin-1 beta (IL-1β), and interleukin-6 (IL-6), thereby alleviating inflammation and tissue injury. It has been shown that Tan IIA can have anti-inflammatory effects by controlling the NF-κB pathways.^[48,49]^ Additionally, it promotes autophagic cancer cell death through the activation of the adenosine monophosphate-activated protein kinase (AMPK) and extracellular signal-regulated kinase signaling pathways against cancer.^[49]^
3. Vasodilation activity: Tan compounds possess vasodilatory effects on blood vessels. They stimulate the release of nitric oxide (NO) through multiple pathways, enhance NO synthesis in endothelial cells, and inhibit the production of vasoconstrictors such as endothelin-1 and angiotensin II thereby promoting further vascular relaxation.^[50]^
4. Anti-thrombotic activity: Tan compounds can inhibit platelet aggregation and coagulation, consequently reducing thrombus formation. They modulate platelet activation signaling pathways, reduce the release of platelet aggregating factors, and increase the production of plasminogen activators, thereby promoting fibrinolysis.^[51,52]^
5. Antitumor activity: Tan compounds exhibit certain antitumor properties. They inhibit tumor cell proliferation, invasion, and metastasis, and induce tumor cell apoptosis. Tan compounds exert their antitumor effects through various mechanisms.^[31,53]^
6. Benefits on cardiovascular and cerebral vascular disease: Tan compounds have potential benefits in the treatment of cardio-cerebral vascular diseases. They can improve cardiovascular and cerebrovascular function through various pathways, including reducing myocardial ischemia/reperfusion injury, decreasing myocardial contractility, inhibiting vascular smooth muscle cell proliferation, and reducing vascular inflammation. Additionally, Tan compounds can promote normal blood flow by improving microcirculation and inhibiting platelet aggregation, thereby reducing the risk of cardiovascular and cerebrovascular diseases. Tan IIA protects the function of cardiac muscle by suppressing reactive oxygen species and modulating the Bcl-2/Bax axis.^[54]^
7. Antidiabetic effects: Studies have shown that Tan compounds have antidiabetic effects. They can lower blood glucose levels, increase insulin sensitivity, and improve pancreatic function by inhibiting diabetes-related inflammatory reactions and oxidative stress.^[55]^ Tan compounds can also inhibit the formation of advanced glycation end-products, reducing the occurrence of diabetic complications.
8. Hepatoprotective effects: Tan compounds have protective effects on liver diseases.^[56]^ They can alleviate liver oxidative stress and inflammatory reactions, inhibit liver fibrosis and hepatocyte apoptosis, thereby improving liver function. Additionally, Tan compounds can promote liver cell regeneration and bile acid synthesis, exerting positive regulatory effects on the liver and biliary system.^[57]^
9. Effects on neurodegenerative diseases: Through antioxidant and anti-inflammatory processes, Tan compounds have demonstrated potential therapeutic benefits in neurodegenerative disorders, which could slow the development of neurodegenerative conditions such as Alzheimer disease and Parkinson disease.^[58]^ Tan compounds also have the ability to inhibit neuronal apoptosis, improve neuronal survival, and promote neuroregeneration, which is important for nerve damage and repair.

#### 3.1.2. The specific mechanism of action of different Tan compounds

These mechanisms of action make Tan compounds play important pharmacological roles in antioxidation, anti-inflammation, vascular protection, anti-thrombosis, and antitumor effects. However, the specific mechanisms of action may vary depending on the different components of Tan compounds. Here are the specific mechanisms of action for several common components of Tan compounds.

##### 3.1.2.1. Tan I and Tan IIA

These 2 compounds are among the most common components in *S miltiorrhiza* and exhibit multiple pharmacological effects. They exert their effects by modulating several signaling pathways, including NF-κB, MAPK, PI3K/Akt, and Nrf2 pathways.^[84–87]^ The regulation of these pathways can inhibit inflammatory responses, reduce cellular oxidative stress, regulate cell growth and apoptosis, thereby exerting antioxidative, anti-inflammatory, antitumor, and cardiovascular and cerebrovascular protective effects. Through altering Nrf2 signals, Tan I has been demonstrated to reduce oxygen consumption and oxidative stress-related cardiomyocyte injury.^[88]^ Additionally, it has been shown to inhibit vascular smooth muscle cell proliferation via insulin-like growth factor 1 receptor/PI3K signal transduction.^[89]^ Tan I can also inhibit the production of vascular endothelial growth factor, cyclin A, and cyclin B, reducing the tumor-activated cell cycle pathway and regulating cell progression in the S phase and G2/M phase.^[90]^

##### 3.1.2.2. Tan IIB

Tan IIB has undergone substantial research because of its anti-inflammatory, antioxidant, anticancer, and neuroprotective effects.^[91]^ It has been found to block the NF-κB signaling mechanism, which lowers the production of inflammatory cytokines. Additionally, it has the capacity to prevent tumor cells from proliferating and invading.^[92]^ Tan IIB has also been demonstrated to regulate the cell cycle and affect the production of proteins linked to apoptosis, making it a potent inhibitor of tumor growth.

### 3.2. *S miltiorrhiza* polysaccharides (SMP)

SMP, an essential active component found in the root of *S miltiorrhiza*, exhibits a wide range of pharmacological effects. These effects can be categorized as follows:

1. Antioxidant activity: SMP demonstrates remarkable antioxidant activity by effectively scavenging free radicals and inhibiting oxidative stress reactions. Through its ability to regulate oxidation-reduction balance, reduce oxidative damage, and inhibit intracellular peroxide production, it offers significant protection against oxidative stress-induced cellular damage.^[59]^ In vitro studies indicate that SMP displays high antioxidant activity with IC50 values of 0.991 mg/mL for DPPH scavenging and 4.007 mg/mL for hydroxyl free radical scavenging. Moreover, it has shown the potential to modulate the activity of antioxidant enzymes in vivo.^[60]^
2. Anti-inflammatory effect: SMP acts as an anti-inflammatory regulator, effectively reducing inflammatory reactions and tissue damage. It achieves this by inhibiting the secretion of inflammatory cytokines such as TNF-α and IL-1β.^[61]^
3. Cardiovascular protection: SMP provides protective effects on the cardiovascular system by exerting various actions. It contributes to the dilation of blood vessels, lowering blood pressure, improving cardiac function, alleviating myocardial ischemia/reperfusion injury, reducing cardiac muscle contractility, and inhibiting the proliferation of vascular smooth muscle cells. Overall, these actions promote normal blood flow.^[62]^ In vitro studies suggest that SMPs inhibit mitochondrial dysfunction, deactivate the caspase-3 cascade, and enhance antioxidant capacity, thus protecting cardiovascular health.^[63]^
4. Antitumor activity: SMP exhibits certain antitumor properties by inhibiting the proliferation, invasion, and metastasis of tumor cells. Additionally, it induces apoptosis in tumor cells. These effects are mediated through various pathways, including the inhibition of angiogenesis, regulation of the cell cycle, and inhibition of tumor-related signaling pathways.^[64]^ Moreover, it has been demonstrated that SMP anticancer action involves the stimulation of natural killer cells and cytotoxic T lymphocytes, as well as the promotion of anti-inflammatory cytokines such as IL-2, IL-4, and IL-10, and the inhibition of pro-inflammatory cytokines like IL-6 and TNF-α.^[65]^
5. Antidiabetic effect: Research indicates that SMP possesses antidiabetic effects.^[66]^ It effectively lowers blood glucose levels, increases insulin sensitivity, and improves pancreatic islet function by suppressing diabetes-related inflammatory reactions and oxidative stress.^[66]^ Furthermore, it exhibits the ability to inhibit the formation of advanced glycation end-products, reducing the occurrence of diabetes-related complications.^[67]^
6. Immunomodulatory effect: SMP possesses immunomodulatory properties, enhancing disease resistance, the body immune response, and the activation and proliferation of immune cells. It also effectively regulates the release of inflammatory mediators and balances immunological responses, making it a potential treatment for immune-related disorders.^[68]^
7. Hepatoprotective effect: SMP demonstrates hepatoprotective properties by alleviating liver oxidative stress, inhibiting inflammatory reactions, and promoting the regeneration of hepatocytes.^[44]^ It also inhibits liver fibrosis and hepatocyte apoptosis, contributing to improved liver function.^[69]^ In addition, it positively regulates the liver and biliary system by promoting hepatocyte regeneration and bile acid synthesis. SMPs aid in the healing of lipopolysaccharide -induced liver injury, as evidenced by the reduction in serum concentrations of alanine aminotransferase, aspartate aminotransferase, and NO.^[44,70]^
8. Neuroprotective effect: SMP exhibits a neuroprotective effect by mitigating the progression of neurodegenerative diseases such as Alzheimer disease and Parkinson disease. It achieves this through its antioxidant and anti-inflammatory mechanisms. Additionally, it inhibits neuronal apoptosis, improves neuronal survival, and promotes neural regeneration, which is crucial for nerve injury and repair.^[71,72]^

### 3.3. Salvianolic acid compounds

Salvianolic acid compounds, including salvianolic acid A (SalA), SalB, and SalC, are active components derived from *S miltiorrhiza*. These compounds are commonly employed in traditional Chinese medicine and have a variety of pharmacological effects, as described below:

1. Antioxidant effect: Salvianolic acid molecules protect cells from oxidative damage by scavenging free radicals and reducing oxidative stress. They can enhance the activity of antioxidant enzymes such as SOD and GPx, thereby mitigating oxidative damage.^[73]^
2. Anti-inflammatory effect: Salvianolic acid molecules can inhibit inflammatory cytokines like TNF-α, IL-1, and IL-6. They also suppress inflammatory signaling pathways, including the NF-kappa B pathway, leading to decreased production and release of inflammatory mediators. Consequently, these compounds alleviate inflammation and tissue damage.^[74]^
3. Cardiovascular protection: Salvianolic acid compounds have multiple protective effects on the cardiovascular system. They promote the release of NO and inhibit the synthesis of endothelin, resulting in vasodilation, reduced blood pressure, and improved blood circulation. Furthermore, they improve cardiac function, increase myocardial contractility, alleviate myocardial ischemia/reperfusion injury, inhibit smooth muscle cell proliferation and migration, and facilitate normal blood flow.^[75–78]^
4. Antiplatelet aggregation effect: Salvianolic acid compounds can inhibit platelet aggregation and thrombus formation, preventing the formation and blockage of blood clots within blood vessels.^[77]^
5. Antitumor effect: Salvianolic acid compounds possess certain antitumor activity. They impede the proliferation, invasion, and metastasis of tumor cells and induce apoptosis. Additionally, they inhibit angiogenesis, regulate the cell cycle, and suppress tumor-related signaling pathways, thus exhibiting an antitumor effect.^[79]^
6. Antibacterial effect: Salvianolic acid compounds demonstrate inhibitory effects against various bacteria, fungi, and viruses, effectively combating infections.^[80]^
7. Immunomodulatory effect: Salvianolic acid compounds have immunomodulatory effects by enhancing immune function, promoting activation and proliferation of immune cells, and improving disease resistance. They also regulate the release of inflammatory mediators, balance immune responses, and hold therapeutic potential in immune-related diseases.^[81,82]^
8. Anti-aging effect: Salvianolic acid compounds can inhibit the generation of free radicals and oxidative stress damage, delaying cellular and tissue aging processes.^[83]^

## 4. Treatment and research progress of *S miltiorrhiza* for DM and its complications

### 4.1. Diabetes mellitus (DM)

Numerous factors have been identified as potential contributors to the variations observed in studies examining the efficacy of *S miltiorrhiza* injections in controlling blood glucose levels. Variations in therapeutic dosage, animal models, and treatment durations have all been found to play a role. For example, a study administering 100 mg/kg of *S miltiorrhiza* injection intraperitoneally to diabetic rats daily for 4 weeks demonstrated reduced blood glucose levels.^[93]^ In contrast, previous studies utilizing diabetic rats did not observe significant changes in blood glucose levels when *S miltiorrhiza* injections were administered at doses of 0.78 mL/kg for a total of 56 days or 0.5 and 1 mL/kg for a 6-week period.^[94,95]^ By activating the CaMKK/AMPK signaling pathway, Qiang et al‘s research team discovered that *S miltiorrhiza* had antidiabetic effects in diabetic animal models by enhancing mitochondrial activity, increasing ATP generation, and reducing MMP.^[96]^ Additionally, *S miltiorrhiza* has been shown to improve insulin resistance and provide protection against the start of T2DM. by minimizing SOD and GPx generation and limiting t-BHP-induced damage to the liver and pancreas.^[66]^

### 4.2. Diabetic nephropathy (DN)

In recent years, there has been growing interest in using *S miltiorrhiza* for the treatment of DN in order to enhance therapeutic outcomes and minimize side effects. Tan IIA, a component of *S miltiorrhiza*, has demonstrated various protective effects against kidney injury in diabetic rats. Chen et al revealed that Tan IIA reduced proteinuria, renal histopathological damage, and malondialdehyde levels while increasing SOD levels in the renal tissues of diabetic rats. Moreover, Tan IIA also decreased levels of inflammatory and fibrosis factors, such as monocyte chemoattractant protein-1, tissue transforming growth factor-β1 (TGF-β1), P-selectin, and C-reactive protein, in the renal cortex and serum. These findings suggest that Tan IIA exerts a protective effect against early renal injury in diabetic rats through its anti-inflammatory, anti-fibrotic, and antioxidant properties.^[97]^ In alignment with this, Li et al conducted bioinformatics analysis and predicted TGF-β1 as a potential key target of Tan IIA for treating diabetic nephropathy. Subsequent studies supported this prediction by demonstrating that Tan IIA significantly reduced TGF-β1 expression levels in high glucose-induced human kidney-2 cells, thereby reducing cell death and improving mRNA expression of TNF-α and IL-6, inflammatory factors. Importantly, overexpression of TGF-β1 reversed the aforementioned effects of Tan IIA, confirming its ability to inhibit high glucose-induced inflammation and pyroptosis in human kidney-2 cells by downregulating TGF-β1.^[98]^

### 4.3. Diabetic retinopathy (DR)

The effects of *S miltiorrhiza* on DR were examined through microscopic observation of retinal tissue in normal mice, DR mice, and mice injected with varying doses of *S miltiorrhiza*. Blood glucose concentration and malondialdehyde content were also measured. The results revealed a significant decrease in the number of microaneurysms in the retina of the DR group, along with a reduction in the number of gangliocytes.^[99]^ Another study demonstrated that the combination of bezafibrate and *S miltiorrhiza* provided protection against DR, potentially by improving vascular leakage and retinal thickness. Furthermore, this combination therapy resulted in a greater reduction in the ratio of oxidized glutathione to reduced glutathione compared to monotherapy.^[100]^

### 4.4. Diabetic peripheral neuropathy

The administration of SalA yielded several positive effects in diabetic rats, including increased paw withdrawal mechanical threshold and motor nerve conduction velocity, decreased deterioration of sciatic nerve pathology, increased AMPK phosphorylation, up-regulated expression of PGC-1, sirtuin 3, and neuronal nitric oxide synthase, without discernible effects on liver kinase B1. These findings suggest that SalA possesses antidiabetic neuropathy effects. Improvements in glucose metabolism via the AMPK-PGC1-sirtuin 3 axis may explain the beneficial effects of SalA on peripheral nerve function in diabetic rats.^[101]^

### 4.5. Macrovascular disease

#### 4.5.1. Coronary heart disease

Among fatalities related to cardiovascular diseases, coronary heart disease is known to have the highest mortality rate.^[102]^ Oxidative stress is considered one of the primary mechanisms contributing to coronary heart disease, as it leads to the accumulation of metabolites and reactive chemicals that cause structural damage and dysfunction in cardiac tissue.^[103]^ A study identified the Nrf2/MAPK signal transduction as one of the oxidative stress mechanisms involved in coronary heart disease. Tan I has the ability to directly target Nrf2, potentially acting as an Nrf2 agonist, while also inhibiting the activation of the MAPK signaling pathway and downstream proteins. This protective action plays a role in safeguarding cardiac tissue and cardiomyocytes.^[39]^ Various proteins, such as inositol-requiring enzyme 1 and activating transcription factor 4, are known to contribute to the increased occurrence of myocardial infarction caused by endoplasmic reticulum stress. Multiple studies have demonstrated that Tan IIA can reduce cardiomyocyte apoptosis in rats by downregulating protein levels in the inositol-requiring enzyme 1 and activating transcription factor 4 pathways.^[104]^

#### 4.5.2. Cerebrovascular disease

Study indicates that the supercritical CO_2_ extract from *S miltiorrhiza* mitigates cerebral ischemic injury by preventing thrombi formation, platelet aggregation, and activation of the PLC/PKC pathway. The content of Tan IIA, Tan I, and cryptotanshinone in supercritical CO_2_ extract from *S miltiorrhiza* is reported to be 57.85%, 5.67%, and 4.55%, respectively.^[105]^ Wang et al attribute the neuroprotective effect of *S miltiorrhiza* against cerebral ischemia injury to its ability to decrease inflammatory markers and activate the Nrf2/HO-1 signaling pathway.^[106]^

#### 4.5.3. Peripheral arterial disease

*S miltiorrhiza* has been shown to induce a dose-dependent vasodilatory response, which is positively correlated. This response occurs through an endothelium-independent mechanism involving internally rectifying K + channels and Ca2 + channels. Additionally, the study observed a significant increase in blood flow and microvessel density in the ischemic limb. These findings corresponded with favorable outcomes in terms of functional limb recovery.^[107]^

## 5. Advantages and limitations of *S miltiorrhiza* and its Future research for DM and its complications

### 5.1. Advantages and limitations of *S miltiorrhiza* in the treatment of DM and its complications

*S miltiorrhiza* contains anti-inflammatory and antioxidant qualities that make it a promising treatment option for DM and its complications. However, there are certain limitations that need to be considered, such as the lack of clinical data, standardization issues, potential drug interactions, and the need for combination therapy. The advantages of *S miltiorrhiza* are as follows: Anti-inflammatory and antioxidant properties: *S miltiorrhiza* contains bioactive compounds like tans and salvianolic acids, which have demonstrated anti-inflammatory and antioxidant effects. These properties can help reduce inflammation and improve β-cell function, thus potentially preventing the progression of DM. Safety and tolerability: Clinical studies have shown that *S miltiorrhiza* is safe and well-tolerated, with few reported side effects. Natural product: With a long history of use in traditional Chinese medicine, *S miltiorrhiza* is a natural product that may appeal to patients who prefer alternative treatments. However, the clinical evidence supporting its efficacy is still limited, and further research is needed to confirm its effectiveness in treating DM and its complications. Other limitations include: Lack of standardization: There is considerable variation in the quality and composition of *S miltiorrhiza* products, making it challenging to compare results between studies and ensure consistent dosing. Potential interactions with other medications: *S miltiorrhiza* may interact with certain medications, particularly blood thinners and antiplatelet drugs, increasing the risk of bleeding. Not a standalone treatment: *S miltiorrhiza* should not be used as a standalone treatment for diabetic nephropathy but rather in combination with other therapies, including lifestyle changes and medications.

### 5.2. Future research direction of *S miltiorrhiza* in the treatment of DM and its complications

*S miltiorrhiza* shows great potential for the treatment of DM and its complications; however, there are several areas that require further attention for future development. One crucial area is standardization, as the variation in product quality and composition hampers result comparison and consistent dosing. Standardizing *S miltiorrhiza* products would enhance the reliability and consistency of clinical studies. Another important aspect is combination therapy. *S miltiorrhiza* has demonstrated effectiveness when used alongside other drugs such as angiotensin-converting enzyme inhibitors, ARBs, and statins. To determine the optimal combinations and dosages of these therapies, additional research is necessary. Understanding the mechanism of action is crucial for developing new and innovative treatments. While the mechanism of action of *S miltiorrhiza* in DM and its complications involves multiple pathways, further research is needed to fully comprehend these mechanisms. Moreover, despite several clinical studies, the evidence supporting the use of *S miltiorrhiza* in the treatment of DM and its complications remains limited. Therefore, more research is required to validate its efficacy. Furthermore, the concept of individualized medicine holds promise for utilizing *S miltiorrhiza* in the treatment of DM. Tailoring therapies based on patients’ genetic, environmental, and lifestyle factors could enhance the effectiveness of *S miltiorrhiza* in the future.

## Acknowledgments

The authors thank the reviewers for their valuable comments in this study.

## Author contributions

**Funding acquisition:** Jinchen Guo.

**Investigation:** Jinxing Liu.

**Writing – original draft:** Jiajie Li.

**Writing– review & editing:** Weibing Shi.
